# Fetal Inferior Vena Cava Thrombosis Associated With Non-Immune Hydrops Fetalis and Maternal Mirror Syndrome

**DOI:** 10.7759/cureus.41357

**Published:** 2023-07-04

**Authors:** Kimberly M Foxworthy, Eneka Lamb, Alexandria Weymon, Emily Roloff, Jessica Garcia De Paredes, Jamie Frost, Vivian C Romero

**Affiliations:** 1 Maternal Fetal Medicine, Michigan State University College of Human Medicine, Grand Rapids, USA; 2 Maternal Fetal Medicine, Corewell Health West, Grand Rapids, USA; 3 Pediatric Radiology, Corewell Health West, Grand Rapids, USA

**Keywords:** fetal ascites, hydrops fetalis, non-immune hydrops fetalis, maternal mirror syndrome, inferior vena cava thrombosis

## Abstract

Prenatal assessment of the inferior vena cava (IVC) should be considered in pregnancies with atypical presentations of fetal ascites and placentomegaly. We examine a case of a 25-year-old gravida 2 para 1 type 1 diabetic female at 29 and 4/7 weeks’ gestation. Ultrasound (US) showed fetal ascites and placentomegaly with increased middle cerebral artery peak systolic velocity (MCA-PSV) suspicious of fetal anemia. Cordocentesis with intrauterine transfusion briefly resolved the fetal ascites, though the mother developed pulmonary edema and pleural effusion, suggestive of mirror syndrome. On US, fetal ascites returned and progressed to non-immune hydrops fetalis, prompting delivery. Neonatal US revealed a heterogenous and calcified thrombus within the IVC.

## Introduction

Fetal thrombosis is a rare condition that most commonly involves the renal veins, inferior vena cava (IVC), and portal vein. The estimated incidence of renal vein and IVC thrombosis is 2.2 cases per 100,000 live births [1]. Prenatal thromboses are infrequent because of well-balanced fetal coagulation and anticoagulation systems [2]. While usually idiopathic, prenatal thromboses may be related to placental vascular processes that cause malperfusion [3]. Additionally, they have been associated with maternal diabetes and congenital thrombophilia [4].

IVC thrombosis is most often diagnosed postnatally. Prenatal diagnosis is made at a median gestational age of 32 weeks [5]. Clinical presentations of prenatal IVC thrombosis include fetal distress, ascites, pleural or pericardial effusion, or non-immune hydrops fetalis (NIHF), which is defined as the accumulation of fluid in at least two or more cavities (pleura, pericardium, peritoneum, or skin) in the fetus in the absence of maternal circulation of red-cell antibodies. The condition may also present as an incidental IVC thrombosis on prenatal ultrasound (US) imaging or might be missed because the evaluation of the IVC is not included in the routine anatomic assessment. Half of the reported cases of IVC thrombosis have been associated with fetal anemia and two-thirds with fetal thrombocytopenia [5]. Neonatal mortality of IVC thrombosis is reported at 23% [6], particularly if hydrops was diagnosed prenatally.

Mirror syndrome, a unique disorder of pregnancy, is characterized by the triad of NIHF, placentomegaly, and maternal edema [7]. The mean gestational age at diagnosis is 27 weeks [8]. Any etiology of NIHF can lead to mirror syndrome. Though the pathogenesis is not fully understood, trophoblastic injury and maternal vascular endothelial dysfunction are thought to play roles in the disorder [9]. Mirror syndrome is associated with increased maternal morbidity and carries a fetal mortality rate of 67% [8].

We describe a unique case of neonatal IVC thrombosis that presented prenatally with fetal ascites and anemia and progressed to maternal mirror syndrome and NIHF with favorable outcomes.

This article was previously presented as an abstract at the 2022 AIUM Annual Meeting on March 13, 2022.

## Case presentation

**Figure 1:**
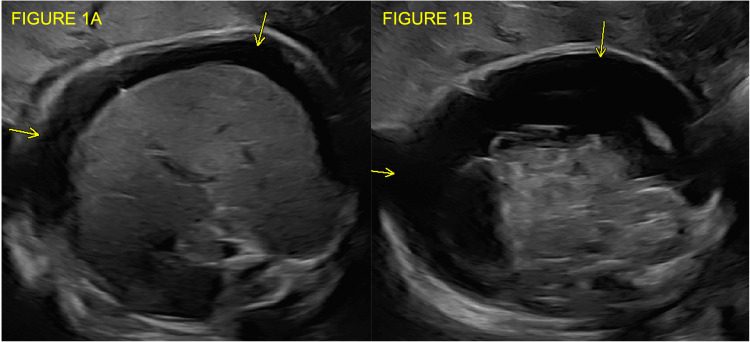
Transverse view of the fetal abdomen at the level of the liver (Figure 1A) and below the liver (Figure 1B) demonstrating fetal ascites (yellow arrows) at 29 and 4/7 weeks' gestation.

A 25-year-old gravida 2 para 1 female presented to the Maternal-Fetal Medicine Department for follow-up US to evaluate fetal interval growth at 29 and 4/7 weeks’ gestation. Her pregnancy was complicated by uncontrolled type 1 diabetes. US showed fetal ascites (Figure 1A, 1B) and an enlarged placenta with a maximum thickness of 7.9 cm. Previous fetal anatomic surveys and fetal echocardiograms had shown no anomalies. Middle cerebral artery peak systolic velocity was elevated at 2.0 MOM, suggesting underlying fetal anemia. The non-stress test showed category one fetal heart rate tracing. The patient was admitted for continuous fetal heart rate monitoring and diagnostic workup for fetal ascites and anemia. Maternal expanded carrier screening was negative and non-invasive prenatal screening results indicated low risk. Maternal blood type was confirmed to be Rh negative with a negative antibody screen. Thyroid function and infectious disease testing were unremarkable. The patient denied any history of blunt trauma or violence that would be concerning for placental abruption.

At 30 and 0/7 weeks’ gestation, percutaneous umbilical blood sampling disclosed a hemoglobin level of 9.8 g/dL and platelet count of 106/µL. An intrauterine transfusion of 85 cc of packed red blood cells and 15 cc of platelets was performed without immediate complications.

**Figure 2:**
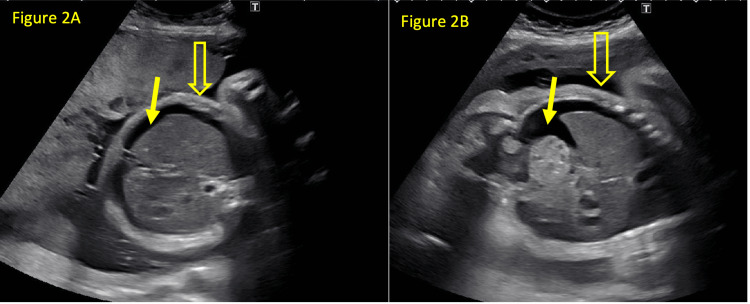
Axial (Figure 2A) and coronal (Figure 2B) views of the fetal abdomen demonstrating recurrent ascites (solid arrow) and skin edema (hollow arrow) at 30 and 3/7 weeks' gestation.

On the day following the intrauterine transfusion, the fetal ascites had been absorbed. Simultaneously, the mother developed dyspnea, right upper quadrant pain, and worsening edema progressing to anasarca. Chest radiography confirmed pulmonary edema and pleural effusion, suggesting mirror syndrome. Treatment with 10 mg intravenous furosemide was initiated. Maternal symptoms progressed despite therapy. Follow-up US at 30 and 3/7 weeks’ gestation revealed recurrent fetal ascites and subcutaneous edema, consistent with NIHF (Figure 2A, 2B). After counseling, the decision was to proceed with cesarean delivery.

**Figure 3:**
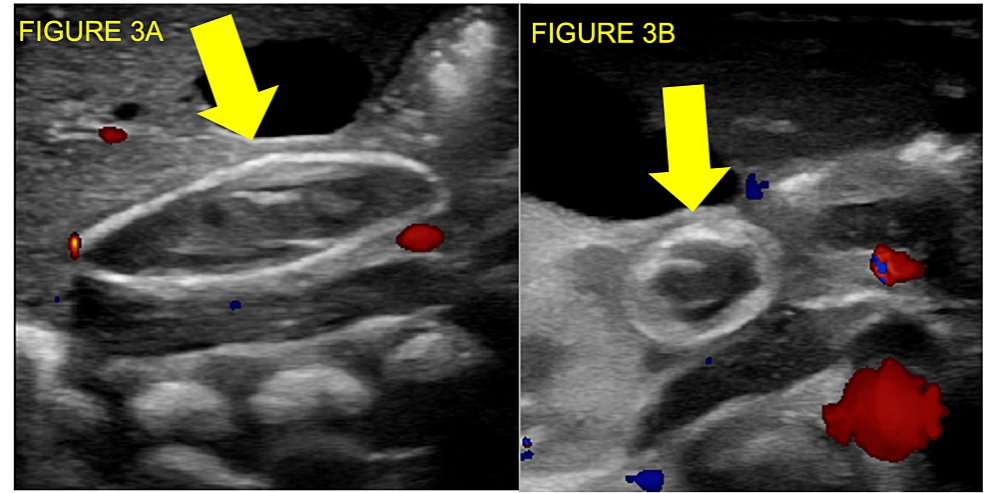
Long axis (Figure 3A) and transverse axis (Figure 3B) ultrasound images on day of life 13 with color Doppler showing a peripherally calcified, expansile, occlusive thrombus (yellow arrows) within the inferior vena cava. Note the persistent ascites.

A 2260g infant was born at 30 and 3/7 weeks’ gestation, with 1- and 5-min APGAR scores of 6 and 9 respectively, arterial cord pH of 7.25, a hemoglobin level of 14.2g/dL, and platelet count of 70x10^3/µL. Paracentesis was performed on the neonate on the day of life one without fluid recovery. Echocardiogram revealed an anomalous large vascular structure adjacent to the upper abdominal aorta. Neonatal abdominal US with Doppler disclosed a heterogenous and calcified thrombus within the IVC at the level of the renal veins, extending into the intrahepatic IVC, with punctate peripheral hepatic echogenicities and significant ascites (Figure 3A, 3B). Renal veins were noted to drain via paraspinal collateral vessels.

Given the stability of the thrombus on repeat US, adequate perfusion of extremities on physical exam, and urine output of approximately 5 mL/kg/hour indicating adequate renal perfusion, the pediatric hematologist recommended against anticoagulation. Hematology follow-up and repeat US every six months were advised.

Maternal symptoms improved on postoperative day one, with a resolution of pulmonary edema on chest radiography. The patient was discharged on postoperative day three after meeting all postpartum milestones.

**Figure 4:**
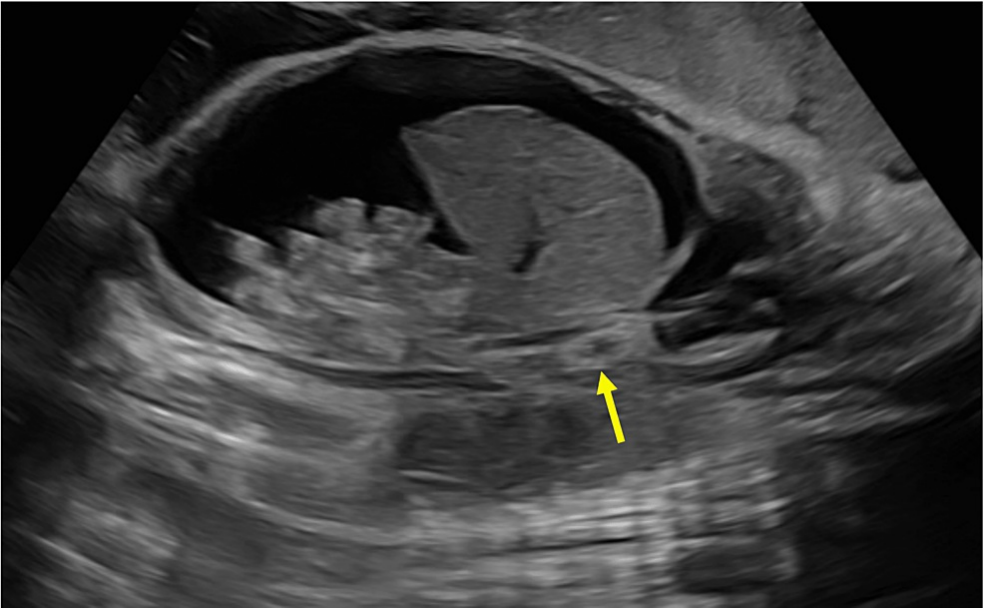
Long axis ultrasound images at 29 and 4/7 weeks' gestation showing a calcified thrombus (yellow arrow) within the inferior vena cava and fetal ascites.

A retrospective review of the US performed at 29 and 4/7 weeks’ gestation was conducted on the day of life 13, and demonstrated the calcified thrombus within the IVC, confirming prenatal IVC thrombosis (Figure 4). Further hematologic workup for an underlying coagulopathy revealed a heterozygous G20210A mutation in the F2 gene.

## Discussion

The etiology of the IVC thrombus initially remained unclear. Thrombus calcification and collateral vessels on postnatal imaging indicated that the thrombus likely occurred in utero, which was confirmed by the retrospective review of the 29 and 4/7 week US. The G20210A mutation identified in our patient causes hyperprothrombinemia that leads to a hypercoagulable state. This condition increases the risk of venous thromboembolism up to fourfold [10]. The mother tested negative for inherited thrombophilia, and the father has not undergone evaluation for this mutation.

IVC thrombosis has been associated with fetal anemia and thrombocytopenia, with a 50% prevalence of the former and 67% of the latter [5]. The proposed mechanism is relatively straightforward; IVC thrombosis consumes erythrocytes and platelets, leading to hemodynamic instability that can result in NIHF, which can in turn cause mirror syndrome. Once mirror syndrome develops, the risk of fetal mortality prompts procedural intervention, to correct either the underlying anemia and/or NIHF to improve fetal survival. The only alternative to improve survival is the induction of labor [8].

Interventions for isolated IVC thrombosis are limited. According to Moaddab et al. [2], there are no reports of prenatal intervention for the closely related condition of renal vein thrombosis. Management focuses instead on surviving neonates and includes anticoagulation, thrombolysis, or conservative follow-up. Interestingly, renal function was normal in all surviving infants in a review of 12 cases [5], as it was in our case.

## Conclusions

Fetal IVC thrombosis is rare and has variable presentations ranging from incidental US findings to fatal NIHF. Our case demonstrates an IVC thrombosis presenting with fetal ascites and elevated MCA Doppler suggesting fetal anemia confirmed by percutaneous umbilical blood sampling. Initial therapy with intrauterine transfusion briefly resolved fetal ascites that recurred and rapidly progressed to maternal mirror syndrome and fetal hydrops, warranting immediate cesarean delivery.

Prenatal evaluation of the fetal IVC is not part of routine obstetric US but might need to be considered when evaluating a fetus with ascites or NIHF to screen for thrombosis. It should also be contemplated in pregnancies with prothrombotic maternal risk factors, such as diabetes, autoimmune disorders, or inherited thrombophilia, particularly in the presence of placentomegaly, fetal ascites, or NIHF.
